# Mobile device use and the cognitive function and depressive symptoms of older adults living in residential care homes

**DOI:** 10.1186/s12877-020-1427-1

**Published:** 2020-02-03

**Authors:** Lu Lin, Xiu-Chen Jing, Shu-Jiao Lv, Jing-Hong Liang, Li Tian, Hui-Ling Li, Martine Puts, Yong Xu

**Affiliations:** 1 The First Affiliated Hospital of Soochow University, Suzhou, China; 2 School of Nursing, Medical College of Soochow University, Suzhou, China; 3 School of Public Health, Medical College of Soochow University, Suzhou, China; 4 Lawrence S. Bloomberg Faculty of Nursing, University of Toronto, Toronto, ON Canada

**Keywords:** Mobile device, Cognitive function, Depressive symptoms, Older adults, Residential care homes

## Abstract

**Background:**

With the fast-paced aging and increasing digitalization of society, there has been a growing interest in the effect of mobile device use on cognitive function and depression in older adults. However, research examining this issue among older adults in residential care homes (RCHs) is scant. Therefore, this study aimed to examine the impact of mobile device use on the cognitive function and depressive symptoms of older adults living in RCHs.

**Methods:**

A cross-sectional survey was conducted using a sociodemographic questionnaire, the Montreal Cognitive Assessment (MoCA) and the 15-item Geriatric Depression Scale (GDS-15).

**Results:**

A total of 235 senior residents (aged 82.58 ± 5.54) in four RCHs were surveyed. Users of mobile devices had a significantly higher total MoCA score (25.02 ± 4.14) and a significantly lower GDS-15 score (3.28 ± 2.74) than non-users (MoCA: 19.34 ± 5.21, GDS-15: 4.69 ± 2.90). Multivariate linear regression indicate that mobile device use is significantly associated with total MoCA score, six of the seven sub-scores (visuospatial abilities and execution functions, attention, language, abstraction, delayed recall, and orientation)(*P* < 0.05). Logistic regression showed that mobile device use was significantly associated with the level of depressive symptoms (OR = 0.458, 95%CI = 0.249–0.845).

**Conclusions:**

Use of mobile devices has a significant association with the cognitive function and depressive symptoms of older adults living in RCHs, and thus should be encouraged as a measure to maintain and improve cognition and prevent depression.

## Background

As the birth rate declines and life expectancy rises, population aging is occurring throughout the world, which is the main driver of the dementia epidemic [1]. It is estimated by the World Alzheimer Report 2018 [2, 3] that there will be a dramatic increase in the number of people living with dementia in the coming decades, from 50 million in 2018 to 152 million by 2050, with a particularly sharp increase in low- and middle-income countries. In 2017, the number of people aged 65 and over in China was 158 million, accounting for 11.4% of the total population [4]. Among them, 4.6% were estimated to have dementia [5]. Mild cognitive impairment (MCI), refers to an intermediate clinical state between normal cognitive aging and dementia [6]. A meta-analysis indicates that the annual conversion rate from MCI to dementia is approximately 20% in community settings and over 30% in memory clinics [7]. It is also noteworthy that depression is often concomitant with cognitive deficits. Population-based studies have found that late-life depression (depression that occurs at the age 60 and above) and its symptoms are associated with cognitive decline, MCI, and dementia [8].

According to a recent systematic review, the estimated prevalence of MCI in China is 14.71% [9]. The prevalence increases with age, ranging from 35 to 80% for people aged 80–105 [10]. This means China is currently faced with the challenge of having to cope with an increasing proportion of its older adults with dementia and MCI. Moreover, there has been an emerging trend of older adults moving from communities to residential care homes for care. The older adults living in residential care homes (RCHs) are generally the middle-old (aged 75–84) and the oldest-old (aged≥85). Age as an intrinsic factor places them at a high risk for cognitive decline, ensuing a high risk of dementia [11, 12], resulting in a higher prevalence of MCI in RCHs than in communities [13].

With the fast-paced aging and increasing digitalization and of society, there has been a growing interest in the effect of mobile device use on the cognitive function of older adults. A study in the UK has revealed that use of mobile devices by older adults can support mental health, promote self-reliance, prevent loneliness, and improve mood [14]. A study in Singapore [15] suggested that compared to those who never or rarely used mobile phones, more frequent (occasional or daily) use of mobile phones was associated with lower rates and risk of decline in global cognitive, attention and working memory, and memory functioning. Barbosa’s study [16] concluded that 3-month use of a communication app on a tablet could enhance social connectedness as well as sense of well-being and confidence with technology among older adults in residential care. However, such research is scant in China.

Therefore, this study aimed to examine the impact of mobile device use on the cognitive performance of institutionalized older adults aged≥65 and their depressive symptoms. The findings of study can provide reference and evidence for the development and implementation of relevant intervention measures in order to slow down the progression of cognitive decline and prevent the occurrence of depressive symptoms, thus reducing the burden on family caregivers, healthcare system and society as a whole.

## Methods

### Study design

An interviewer-administered survey was used.

### Participants

In this study, four residential care homes in Suzhou were selected by convenience sampling and all the senior residents meeting the following inclusion criteria were surveyed: (1) aged ≥65 years; (2) a resident of the RCH for≥3 months; 3) willing to participate in the survey and able to sign the informed consent. Exclusion criteria included: (1) cerebral diseases caused by various underlying medical conditions; (2) acute onset of diseases within the past 3 months, e.g. stroke, heart attack, asthma attack, acute pneumonia, acute bronchitis, etc.; (3) a diagnosis of active epilepsy; (4) a diagnosis of dementia; (5) severe sensory impairments, making them unable to complete cognitive assessment test. The above-mentioned conditions were diagnosed by a doctor.

### Instruments

#### Sociodemographic questionnaire

A sociodemographic questionnaire was used, consisting of key influencing factors for cognitive functioning in older adults extracted from relevant literature, i.e. age, sex, height and weight (converted into body mass index (BMI) by the interviewer on site), level of education (elementary school and below, junior high school, senior high school/secondary school, college and above), monthly income (<5000RMB, 5000-10000RMB, >10000RMB, frequency of socialization (Never, ≤3 times/week, > 3 times/week), type of underlying diseases (categorized into “none” or “cardiovascular/cerebrovascular diseases and/or diabetes” or “others” during statistical analyses.), and use of mobile devices (users, non-users). Mobile devices refer to any handheld computer or smartphone, including smartphones, tablets, e-readers, personal digital assistants (PDAs) and portable music/video players with smart capabilities and Internet access. The participants were asked whether they possessed a mobile device and what they used it for. If they possessed a device and used the smart capabilities and Internet access of the device for communication, entertainment and information search, they were categorized as “users of mobile devices”. If they did not possess a device or they possessed a device but did not use it/did not know how to use it (e.g. they used a smartphone in the same way as a feature phone), they were classified as “non-users”.

#### Montreal cognitive assessment (MoCA)

Use of the MoCA is recommended by the “Guidelines for the diagnosis and treatment of dementia and cognitive impairment in China” [17] and “Expert Consensus on Memory Examination in China” [18]. MoCA is now available in several Chinese versions including Beijing, Guangdong, and Changsha. The Beijing version has a sensitivity of 0.92, a specificity of 0.84, a test-retest reliability coefficient of 0.86, and an internal consistency reliability Cronbach α coefficient of 0.82 [19], and previous research [20] indicates that it is more suitable for assessing the cognitive function of older adults in Suzhou. According to the study conducted by Zhang et al. in nursing homes in southern China, the range of MoCA score for MCI among institutionalized older adults is 15–24 points [21], which was used in our study.

#### The 15-item geriatric depression Scale-15 (GDS-15)

The Chinese version of the 15-item Geriatric Depression Scale (GDS-15) was used. It has been validated in Chinese populations with a test-retest reliability coefficient of 0.728 and a Cronbach’s α coefficient of 0.79 for a score range of 0–15 [22]. The recommended cut-off score of 5 was used in our study to identify participants with depressive symptoms (GDS-15 Score ≥ 5) and those without (GDS-15 Score < 5) [23]. Higher scores indicate more depressive symptoms are present.

#### Procedures

Telephone invitations of the survey were made to all eligible residents of the four selected RCHs. Only those who consented to participate and signed a written informed consent were interviewed. The sociodemographic information questionnaire, MoCA and GDS-15 were filled out by trained researchers in an electronic form using a tablet or smartphone on site while interviewing participants. Two researchers were paired together - one was responsible for interviewing the patient and filling out the form, and the other was responsible for overseeing the input of data. They switched positions every other participant. The survey was conducted from January, 2017 to December, 2017.

### Statistical analysis

SPSS version 23.0 software was used for statistical analyses. Univariate analysis was used to determine whether there was a significant difference between the mobile device users and non-users living in RCHs in the total MoCA score, the sub-scores of each dimension and the GDS-15 score. Parametric statistical tests were performed for normally distributed data and non-parametric tests for abnormally distributed data. After testing the assumptions for multivariate regression such as presence of linearity between the independent variables and the dependent variable and the absence of multicollinearity between the independent variables, a multivariate linear regression model was used to control for confounding factors such as age, sex, BMI, level of education, monthly income, frequency of socialization and underlying diseases. Mobile device use, age, sex, BMI, level of education, monthly income, frequency of socialization and type of underlying disease were used as the independent variables, and the total MoCA score and the sub-scores of each dimension as dependent variables respectively to analyze the impact of mobile device use on cognitive function. A binary logistic regression model was used to examine the impact of mobile device use on the level of depressive symptoms while adjusting for the confounding factors age, sex, BMI, level of education, monthly income, frequency of socialization and type of underlying disease. In the model, absence of depressive symptoms (GDS-15 score < 5) was assigned the value of “0” while presence of depressive symptoms was assigned the value of “1” (GDS-15 score ≥ 5). *P* < 0.05 indicates a statistically significant difference.

## Results

### Sociodemographic information of the older adults living in RCHs

Of the 465 residents invited, a total of 235 senior residents (50.5%) who had been institutionalized for more than 3 months agreed to participate in the study, including 97 male residents (41.3%) and 138 female residents (58.7%), mean age 82.58 ± 5.54 years old, 98 mobile device users (41.7%, mean age 81.19 ± 0.50) and 137 non-users (58.3%, mean age 83.57 ± 0.49). The prevalence of MCI and depressive symptoms was 32.8% and 37.9% respectively. There is a significantly higher percentage of MCI and depressive symptoms in non-users (26.4% and 27.2% respectively) than that in mobile device users (6.4% and 10.6% respectively). For a detailed description of the sample, see Table 1.

**Table 1:** Mobile device users versus non-users in sociodemographics, MCI and depressive symptoms (*N* = 235)

Item	N(%)	Mobile Device Users N(%)	Non-users N(%)	*t*	*P*
Age				3.946	< 0.001
65–74	19(8.1)	10(4.3)	9(3.8)		
75–84	139(59.1)	69(29.4)	70(29.8)		
85–89	52(22.1)	15(6.4)	37(15.7)		
≥ 90	25(10.7)	4(1.7)	21(8.9)		
Sex				−0.656	0.512
Male	97(41.3)	38(16.2)	59(25.1)		
Female	138(58.7)	60(25.5)	78(33.2)		
Education				−5.075	< 0.001
Elementary school and below	35(14.9)	5(2.1)	30(12.8)		
Junior high school	37(15.7)	9(3.8)	28(11.9)		
Senior high school/Secondary school	61(26.0)	29(12.3)	32(13.6)		
College and above	102(43.4)	55(23.4)	47(20.0)		
BMI				−0.889	0.375
< 18.5	25(10.6)	7(3.0)	18(7.7)		
18.5–23.9	129(54.9)	57(24.3)	72(30.6)		
24–28	63(26.8)	25(10.6)	38(16.2)		
> 28	18(7.7)	9(3.8)	9(3.8)		
Monthly Income				−2.243	0.026
< 5000RMB	86(36.6)	26(11.1)	60(25.5)		
5000-10000RMB	134(57.0)	66(28.1)	68(28.9)		
> 10000RMB	15(6.4)	6(2.6)	9(3.8)		
Socialization				−4.564	< 0.001
Never	80(34.1)	14(6.0)	66(28.1)		
≤3 times/week	88(37.4)	50(21.2)	32(16.2)		
> 3 times/week	67(28.5)	34(14.5)	33(14.0)		
Underlying Disease				0.562	0.575
None	23(9.8)	10(4.3)	13(5.5)		
Cardiovascular/cerebrovascular diseases and/or diabetes	202(86.0)	85(36.2)	117(49.8)		
Others	10(4.3)	3(1.3)	7(3.0)		
MCI (MoCA Score 15–24)	77(32.8)	15(6.4)	62(26.4)	7.244	< 0.001
Depressive Symptoms (GDS-15 Score ≥ 5)	89(37.9)	25(10.6)	64(27.2)	3.445	0.001
Total	235(100)	98(41.7)	137(58.3)		

*MCI Mild Cognitive Impairment, *GDS-15* The 15-item Geriatric Depression Scale, *MoCA* Montreal Cognitive Assessment

### Mobile device users vs. non-users in MoCA scores

Comparing the total MoCA score of mobile device users with that of non-users, the overall MoCA scores of the two groups were 25.02 ± 4.14 and 19.34 ± 5.21 respectively, the difference being statistically significant (t = − 9.313, *P* < 0.001). The users of mobile devices had significantly higher scores than non-users in all the seven sub-scores, namely, visuospatial abilities and executive functions (4.09 ± 1.29 vs. 2.62 ± 1.44, *P* < 0.001), naming (2.84 ± 0.40 vs. 2.66 ± 0.68, *P* < 0.05), attention (5.54 ± 0.95 vs. 4.86 ± 1.42, *P* < 0.001), language (1.95 ± 0.91 vs. 1.33 ± 0.85, P < 0.001), abstraction (1.46 ± 0.66 vs. 0.94 ± 0.82, P < 0.001), delayed recall (2.97 ± 1.63 vs. 1.20 ± 1.49, P < 0.001) and orientation (5.73 ± 0.75 vs. 5.05 ± 1.46, P < 0.001). See Table 2 for the full results.

**Table 2:** MoCA scores among mobile device users versus non-users

Use of Mobile Devices	N (%)	Visuospatial/Executive	Naming	Attention	Language	Abstraction	Delayed Recall	Orientation	Total MoCA Score
NO	137 (58.3)	2.62 ± 1.44	2.66 ± 0.68	4.86 ± 1.42	1.33 ± 0.85	0.94 ± 0.82	1.20 ± 1.49	5.05 ± 1.46	19.34 ± 5.21
YES	98 (41.7)	4.09 ± 1.29	2.84 ± 0.40	5.54 ± 0.95	1.95 ± 0.91	1.46 ± 0.66	2.97 ± 1.63	5.73 ± 0.75	25.02 ± 4.14
*t*		−8.225	−2.445	−4.341	−5.352	−5.161	−8.612	−4.673	−9.313
*P*		< 0.001	0.015	< 0.001	< 0.001	< 0.001	< 0.001	< 0.001	< 0.001

*MoCA Montreal cognitive assessment

In multivariate linear regression analysis, after controlling for factors such as age, sex, BMI, level of education, monthly income, frequency of socialization and underlying disease, use of mobile devices was found to be significantly associated with the total MoCA score and six of the seven sub-scores, i.e. visuospatial abilities and execution functions, attention, language, abstraction (*P* < 0.001), delayed recall, and orientation (*P* < 0.01); however, no such association was found in the dimension of naming (*P* > 0.05). See Table 3.

**Table 3:** Multivariate linear regression analyses of mobile device use and MoCA scores

Dependent Variables	Beta	*SE*	*P*	95%CI
Visuospatial/Executive	0.340	0.190	< 0.001	0.696, 1.445
Naming	0.079	0.082	0.339	−0.083, 0.241
Attention	0.488	0.181	0.008	0.131, 0.844
Language	0.432	0.126	0.001	0.184, 0.681
Abstraction	0.304	0.104	0.004	0.100, 0.508
Delayed Recall	1.475	0.224	< 0.001	1.034, 1.951
Orientation	0.603	0.177	0.001	0.254, 0.953
Total MOCA Score	4.454	0.685	< 0.001	3.104, 5.804

*MoCA Montreal cognitive assessment

### Mobile device users vs. non-users in GDS-15 score

The GDS-15 scores of mobile device users (*n* = 98) and non-users (*n* = 137) were 3.28 ± 2.74 and 4.69 ± 2.90 respectively, presenting a statistically significant difference (*Z* = -4.158, *P* < 0.001) as shown by the results of univariate analysis. After adjusting for age, sex, BMI, level of education, monthly income, frequency of socialization, and type of underlying disease in the logistic regression model (1 = depressive symptoms present), use of mobile devices was found to be negatively correlated with depressive symptoms (OR = 0.458, 95%CI = 0.249–0.845). See Table 4.

**Table 4:** Logistic regression analysis of mobile device use and depressive symptoms

Covariates	*β*	*SE*	*Waldχ*^*2*^	*P*	OR	95%CI	
Age			3.735	0.292			
Age(1)	1.021	0.655	2.426	0.119	2.775	0.768, 10.026	
Age(2)	1.216	0.712	2.917	0.088	3.375	0.836, 13.627	
Age(3)	0.554	0.770	0.517	0.472	1.740	0.385, 7.868	
Sex(1)	−0.843	0.314	7.194	0.007	0.430	0.232, 0.797	
BMI			2.554	0.466			
BMI(1)	−0.279	0.502	0.309	0.578	0.756	0.283, 2.024	
BMI(2)	0.273	0.541	0.254	0.614	1.314	0.455, 3.795	
BMI(3)	0.069	0.726	0.009	0.924	1.072	0.258, 4.443	
Level of Education			3.890	0.274			
Level of Education(1)	−0.280	0.521	0.289	0.591	0.756	0.272, 2.099	
Level of Education(2)	−0.984	0.522	3.545	0.060	0.374	0.134, 1.041	
Level of Education(3)	−0.616	0.489	1.590	0.207	0.540	0.207, 1.407	
Monthly Income			0.538	0.764			
Monthly Income(1)	−0.259	0.365	0.503	0.478	0.772	0.377, 1.579	
Monthly Income(2)	−0.272	0.643	0.179	0.672	0.762	0.216, 2.685	
Socialization			1.402	0.496			
Socialization(1)	−0.177	0.367	0.232	0.630	0.838	0.408, 1.721	
Socialization(2)	−0.464	0.394	1.384	0.240	0.629	0.290, 1.362	
Underlying Diseases			2.216	0.330			
Underlying Diseases(1)	0.819	0.550	2.215	0.137	2.268	0.771, 6.666	
Underlying Diseases(2)	0.704	0.867	0.659	0.417	2.022	0.369, 11.073	
Mobile Device Use(1)	−0.681	0.340	4.013	0.045	0.506	0.260, 0.985	
Constant	−0.464	0.963	0.232	0.630	0.629		

*Depressive symptoms present = 1

*Age (0):65–74, Age (1):75–84, Age (2):85–89, Age (3):≥90

Sex (0):Male, Sex(1):Female

BMI(0):< 18.5, BMI(1):18.5–23.9, BMI(2):24–28, BMI(3):> 28

Education(0):Elementary school and below, Junior high school; Education(1):Senior high school/Secondary school; Education(2):College and above

Monthly Income(0):<5000RMB, Monthly Income(1):5000-10000RMB, Monthly Income(2): >10000RMB

Socialization(0): Never, Socialization(1): ≦3 times/week, Socialization(2): > 3 times/week

Underlying Disease(0): None, Underlying Disease(1): Cardiovascular/cerebrovascular diseases and/or diabetes, Underlying Disease(2): Others

Mobile Device Use(0): No, Mobile Device Use(1): Yes

## Discussion

The results of our study reveal that mobile device use is significantly associated with total MoCA score, six of the seven sub-scores (visuospatial abilities and execution functions, attention, language, abstraction, delayed recall, and orientation) and GDS-15 score in residents of RCHs (*P* < 0.05). Social interaction is considered as one of the lifestyle factors that play a significant role in maintaining or improving cognitive functioning in older adults. A growing body of literature has described positive effects of social engagement and negative effects of social isolation on cognitive performance in older adults [24]. Previous research [25, 26] has shown that frail older adults living in retirement homes are especially vulnerable to social isolation and loneliness, which is related to decreased social networks, mobility, health status, and interaction with close ties. The most distinctive features of mobile devices are access to Internet, multimedia and multi-task processing. In our study, the mobile devices users living in RCHs used the device for communication with social ties (family members, friends, staff in the RCH, etc.), entertainment (listening to music, watching videos, playing games, etc.) and information search (keeping up with current events and latest news, looking up recipes and health information online, etc.). Studies suggest that digital communication technologies embedded in mobile devices can contribute to social connectedness (meaningful social interaction) and help tackle feelings of loneliness and factors leading to social isolation in later life [27, 28]. Enhanced social connectedness may have contributed to the significantly higher cognitive scores of mobile device users in MoCA test and lower risks for geriatric depression in our study. The results of the logistic regression have revealed that the female residents in RCHs had a significantly lower risk for depressive symptoms, which might be due to that they were more socially engaged than male residents.

Research by Small et al. [29] points out that Internet use augments cognitive skills, stating that some areas of the brain in the 24 neurologically normal subjects aged 55–76 were more active in both decision-making tasks and complex reasoning tasks after several sessions of Internet search. These results indicate that, in spite of the changes in neurobiological functioning caused by aging, complex cognitively-engaging activities such as Internet searching may improve brain functions in older adults, thus enhancing their cognitive skills, especially executive functions. In addition, playing games on mobile devices has also been proven to have generalized positive effects on cognitive control abilities in older adults [30]. These findings are consistent with the results of our survey—mobile device users performed significantly better in dimensions such as visuospatial abilities and execution functions, attention and orientation than non-users.

A meta-analysis revealed that the pooled prevalence of depressive symptoms in Chinese older adults was 23.6% [31]. The prevalence of depressive symptoms among the RCH residents in our study was 37.9%, which is consistent with the findings of Jongenelis et al. [32] that the prevalence of depression in the residential care population is higher than that in the community-setting. Studies have revealed that self-confidence, feelings of loneliness, social interaction, satisfaction with one’s life, and depression can be improved when older adults learn how to use computers and gain access to Internet [33, 34]. Moreover, older adults are motivated to use digital technology to support their mental health through mechanisms of distraction (from unwanted thoughts and may help to concentrate the mind), normalization (reading online descriptions of symptoms of poor mental health are felt to be normalizing), and facilitated expression of mental states (helpful for exploring their own feelings and being able to express these) [14]. These findings suggest a positive effect of mobile device use on the mental well-being of older adults [16], which is also manifested by our research results with the mobile device users having a significantly lower GDS-15 score than the non-users.

However, the effects of mobile device use on everyday cognitive functioning are mixed [35]. The problems remain how to maximize its beneficial effects and minimize its adverse effects. After adjusting for multiple variables such as age, sex, BMI, education, etc., the results of this study show that use of mobile devices is an independent factor that influences the cognitive function in older adults living in RCHs as it is statistically significant in multivariate linear regression models of total MoCA score and the six sub-scores, namely, visuospatial abilities and executive functions, attention, language and abstraction, delayed recall, orientation. The reasons might be: (1) older adults that use mobile devices are willing to embrace new things and are more confident with new technology—such open-mindedness and optimism benefits cognition to some extent; (2) using instant messaging apps such as WeChat or QQ on the mobile devices strengthens their ties with families and friends and improves social-connectedness, which in turn enhances cognitive function; (3) using mobile devices for entertainment and information search is a source of constant cognitive stimulation. Nevertheless, considering the fact that a considerable proportion of older adults suffer from vision impairment and hearing loss, they should be trained as how to moderately use the mobile device for cognitive enhancement without worsening their existing health problems.

There are several limitations to the present study. Firstly, this study was a cross-sectional survey, and thus it is unclear what the relationship between mobile device use and cognitive function is-whether use of mobile devices protect against cognitive decline or those who have cognitive decline are no longer able to use their device. Secondly, the habit of mobile device use (i.e. for how long the older adults have been using the device, how often they use the device, how much time they spend using it each day, etc.) was not discussed in our study, which can be taken into consideration by future research. Thirdly, comorbidities that could affect cognitive functioning and mood, such as vitamin B12 and D depletions, hypothyroidism were not considered in our study. Fourthly, the diagnosis of MCI was based on the MoCA score. Therefore, multi-center studies with a larger sample size should be carried out in the future to further examine the effect of mobile device use on cognitive function and depression in residents of RCHs as well as the cognitive trajectory of mobile device users over time of usage and time of institutionalization. In future studies, the size of the resident’s social network, the type of activities they perform throughout the day in RCHs and the type of activities performed with the mobile device and the frequency of using the device should also be taken into account.

## Conclusions

Use of mobile devices has a significant association with the cognitive function and depressive symptoms of older adults living in RCHs, and thus proper device use should be encouraged as a measure to maintain and improve cognitive functioning, reduce social isolation and enhance social connectedness, and prevent the occurrence of depressive symptoms.

## Data Availability

The datasets used and/or analyzed during the current study are available from the corresponding author on reasonable request.

## Abbreviations

BMI: Body mass index
GDS-15: The 15-item geriatric depression scale
MCI: Mild cognitive impairment
MoCA: The Montreal cognitive assessment
PDAs: Personal digital assistants
RCHs: Residential care homes

## References

[1] Prince M, Bryce R, Albanese E, Wimo A, Ribeiro W (2013). Ferri CP. Alzheimers Dement.

[2] Patterson C (2018). World Alzheimer report 2018.

[3] Liang JH, Shen WT (2019). The optimal treatment for improving cognitive function in elder people with mild cognitive impairment incorporating Bayesian network meta-analysis and systematic review. Ageing Res Rev.

[4] National Bureau of Statistics of China (2018). Statistical communiqué of the People’s republic of China on the 2017 national economic and social development.

[5] Wu YT, Lee HY, Norton S, Chen C (2013). Prevalence studies of dementia in mainland China, Hong Kong and Taiwan: a systematic review and meta-analysis. PLoS One.

[6] Petersen RC, Smith G, Waring S (1999). Mild cognitive impairment. Clinical characterization and outcome. Arch Neurol.

[7] Mitchell AJ, Shiri-Feshki M (2009). Rate of progression of mild cognitive impairment to dementia - meta-analysis of 41 robust inception cohort studies. Acta Psychiatr Scand.

[8] Wei J, Ying M (2019). Late-life depression and cognitive function among older adults in the U.S.: the National Health and nutrition examination survey, 2011-2014. J Psychiatr Res.

[9] Xue J, Li J (2018). The prevalence of mild cognitive impairment in China: a systematic review. Aging Dis.

[10] Zeng Y, Feng Q (2017). Survival, disabilities in activities of daily living, and physical and cognitive functioning among the oldest-old in China: a cohort study. Lancet.

[11] Zhang YD, Xu Y (2010). A meta-analysis of risk factors for senile dementia in Chinese population. Chin J Gerontol.

[12] Prince M, Wimo A, Guerchet M, Ali GC, Wu YT, Prina AM (2015). The global impact of dementia: an analysis of prevalence, incidence, cost and trends.

[13] Qui Q, Wang YJ (2019). interRAI-LTCF-assessed cognitive impairment and influencing factors in elderly people in geriatric nursing facilities. Chinese Gen Pract.

[14] Andrews JA, Brown LJ (2019). Older adults’ perspectives on using digital technology to maintain good mental health: interactive group study. J Med Internet Res.

[15] Ng TP, Lim ML (2012). Long-term digital mobile phone use and cognitive decline in the elderly. Bioelectromagnetics..

[16] Barbosa NB, Franz R (2019). Can digital technology enhance social connectedness among older adults? A Feasibility Study. J Appl Gerontol.

[17] Committee for Cognitive Disorders under Neurology Physician Branch of Chinese Medical Doctor Association (2018). 2018 guidelines for the diagnosis and treatment of dementia and cognitive impairment in China (V): diagnosis and treatment for mild cognitive impairment. Natl Med J China.

[18] Xie HG, Tian JZ, Wang LN (2014). Expert consensus on memory examination in China. Chinese J Int Med.

[19] Zhang YD (2011). Status quo, risk factors and early intervention of mild cognitive impairment in the elderly.

[20] Zhang JA (2013). An investigation and analysis of mild cognitive impairment in the elderly population in Taicang City.

[21] Zhang LX, Liu XQ (2008). Determination of the cut-off point of the Chinese version of the Montreal cognitive assessment among Chinese elderly in Guangzhou. Chin Ment Health J.

[22] Tang D (2013). Application of short form geriatric depression scale (GDS-15) in Chinese elderly. Chinese J Clin Psychol.

[23] Pocklington C (2016). The diagnostic accuracy of brief versions of the geriatric depression scale: a systematic review and meta-analysis. Int J Geriatr Psychiatry.

[24] Myhre JW, Mehl MR (2017). Cognitive benefits of online social networking for healthy older adults. J Gerontol B Psychol Sci Soc Sci.

[25] Prieto-Flores ME, Forjaz MJ, Fernandez-Mayoralas G, Rojo-Perez F, Martinez-Martin P (2011). Factors associated with loneliness of noninstitutionalized and institutionalized older adults. J Aging Health.

[26] Victor C, Scambler S, Bond J (2009). The social world of older people: understanding loneliness and social isolation in later life.

[27] Khosravi P, Rezvani A, Wiewiora A (2016). The impact of technology on older adults’ social isolation. Comput Hum Behav.

[28] Masi C, Chen HY, Hawkley L, Cacioppo JT (2011). A meta-analysis of interventions to reduce loneliness. Personal Soc Psychol Rev.

[29] Small GW, Moody TD, Siddarth P, Bookheimer SY (2009). Your brain on Google: patterns of cerebral activation during internet searching. Am J Geriatr Psychiatry.

[30] Anguera JA, Boccanfuso J (2013). Video game training enhances cognitive control in older adults. Nature..

[31] Li D (2014). A meta-analysis of the prevalence of depressive symptoms in Chinese older adults. Arch Gerontol Geriatr.

[32] Jongenelis K, Pot A, Eisses A, Beekman A, Kluiter H, Ribbe M (2004). Prevalence and risk indicators of depression in elderly nursing home patients: the AGED study. J Affect Disord.

[33] Shapira N, Barak A, Gal I (2007). Promoting older adults’ well-being through internet training and use. J Aging Ment Health.

[34] Ordonez TN, Yassuda MS (2011). Elderly online: effects of a digital inclusion program in cognitive performance. Arch Gerontol Geriatr.

[35] Wilmer HH, Sherman LE (2017). Smartphones and cognition: a review of research exploring the links between Mobile technology habits and cognitive functioning. Front Psychol.

